# Cardiovascular Risk Factors, Alzheimer’s Disease, and the MIND Diet: A Narrative Review from Molecular Mechanisms to Clinical Outcomes

**DOI:** 10.3390/nu17142328

**Published:** 2025-07-16

**Authors:** Amirhossein Ataei Kachouei, Saiful Singar, Amber Wood, Jason D. Flatt, Sara K. Rosenkranz, Richard R. Rosenkranz, Neda S. Akhavan

**Affiliations:** 1 Faculty of Life Sciences: Food, Nutrition and Health, University of Bayreuth, 95326 Kulmbach, Germany; amirhossein.ataei-kachouei@uni-bayreuth.de; 2 Department of Health, Nutrition, and Food Sciences, Anne Spencer Daves College of Education, Health, and Human Sciences, Florida State University, Tallahassee, FL 32306, USA; ssingar@fsu.edu; 3 Department of Kinesiology and Nutrition Sciences, School of Integrated Health Sciences, University of Nevada Las Vegas, Las Vegas, NV 89154, USA; wooda13@unlv.nevada.edu (A.W.); sara.rosenkranz@unlv.edu (S.K.R.); richard.rosenkranz@unlv.edu (R.R.R.); 4 Department of Social and Behavioral Health, School of Public Health, University of Nevada Las Vegas, Las Vegas, NV 89154, USA; jason.flatt@unlv.edu

**Keywords:** dietary interventions, hypertension, hyperlipidemia, diabetes mellitus, obesity, amyloid-β

## Abstract

Cardiovascular diseases (CVDs) and Alzheimer’s disease (AD) are among the top 10 causes of death worldwide. Accumulating evidence suggests connections between CVD risk factors―including hypertension (HTN), hyperlipidemia (HLP), diabetes mellitus (DM), obesity, and physical inactivity―and AD. The Mediterranean–DASH Intervention for Neurodegenerative Delay (MIND) dietary pattern has recently garnered considerable attention as a key preventive strategy for both CVDs and AD. While previous studies have examined the connections between CVD risk factors and AD, they have not thoroughly explored their underlying mechanisms. Therefore, the current literature review aims to synthesize the literature and highlight underlying mechanisms from preclinical to clinical studies to elucidate the relationship between CVD risk factors, AD, and the role of the MIND dietary pattern in these conditions. The MIND dietary pattern emphasizes foods rich in antioxidants and brain-healthy nutrients such as vitamin E, folate, polyphenols, flavonoids, carotenoids, fiber, monounsaturated fatty acids, and omega-3 fatty acids. These components have been associated with reduced amyloid-β accumulation in preclinical studies and may contribute to the prevention of AD, either directly or indirectly by affecting CVD risk factors. Despite the extensive evidence from preclinical and observational studies, few clinical trials have investigated the effects of the MIND dietary pattern on cognitive health. Therefore, long-term clinical trials are required to better understand and establish the potential role of the MIND dietary pattern in preventing and managing AD.

## 1. Background

Cardiovascular diseases (CVDs) remain the leading cause of morbidity and mortality in the United States, with approximately 1 million annual deaths [1,2]. Annual healthcare costs associated with CVDs represent a large economic burden, which is projected to quadruple (from USD 393 billion to 1490 billion) and triple (from USD 400 billion to USD 1344 billion) between 2020 and 2050, respectively [3]. Both modifiable and non-modifiable risk factors contribute to the etiology of CVDs, with non-modifiable risk factors including age, biological sex, ethnicity, and family history of CVDs, and common modifiable risk factors including hypertension (HTN), hyperlipidemia, diabetes, obesity, physical inactivity, and smoking [4].

The relationship between CVD risk factors and the development of CVDs is complex, attributable to various pathways and mechanisms [5]. For example, a dysfunctional endothelium, characterized by reduced vasodilation, increased proliferation of vascular smooth muscle cells, and a proinflammatory/prothrombotic state, greatly contributes to CVD risk factors, including HTN, hypercholesterolemia, and insulin resistance [6]. Additionally, elevated oxidative stress―which can occur as a result of poor dietary intake, hyperlipidemia, smoking, insulin resistance, obesity, and stress―contributes to endothelial dysfunction and vascular damage in the pathogenesis of CVD [6,7]. Collectively, these mechanisms play crucial roles in the pathophysiological processes that lead to the development of atherosclerosis [8,9].

Noticeably, previous epidemiological studies have shown that CVDs and Alzheimer’s disease (AD) share common risk factors [10,11,12]. AD, the most common type of dementia, currently affects approximately 6.9 million Americans aged 65 and older, a number projected to rise to 13.5 million by 2026 [13]. AD is the fifth leading cause of death in adults over the age of 65 in the U.S. and is another significant contributor to economic burden, with the cost of health care for older adults with dementia estimated to be USD 360 billion in 2024 [13].

CVDs and AD are interconnected through multiple biological mechanisms, such as endothelial dysfunction, oxidative stress, inflammation, and disruption of the blood–brain barrier (BBB) (Table 1). For example, chronic HTN and hypercholesterolemia can lead to endothelial dysfunction, which is associated with increased levels of amyloid-β (Aβ) and tau protein—key biomarkers of AD [14,15,16]. Furthermore, oxidative stress and inflammation, which contribute to CVDs through vascular damage, atherosclerosis, and impaired nitric oxide (NO) signaling, also promote amyloidogenesis and tau hyperphosphorylation [17,18,19]. The accumulation of Aβ around neurons and the hyperphosphorylation of tau protein ultimately result in the formation of amyloid plaques and neurofibrillary tangles (NFTs), respectively, which are central to the predominantly hypothesized pathogenesis of AD [20]. Therefore, considering the associations between CVDs and AD, a comprehensive prevention strategy that could simultaneously reduce the risk of both conditions is of great interest.

It has been well documented that healthy dietary patterns play a significant role in preventing and managing CVD risk factors [21,22,23]. This is particularly true for dietary patterns rich in fruits, vegetables, whole grains, nuts, seeds, and legumes [23]. The Mediterranean dietary pattern is known for its emphasis on olive oil, vegetables, fruits, cereals, nuts, fish, and pulses/legumes, along with a moderate intake of red meats, dairy products, and red wine. The Dietary Approaches to Stop Hypertension (DASH) diet is another well-established plant-based dietary pattern that focuses on a high intake of fruits, vegetables, nuts, seeds, legumes, lean meats, fish, poultry, and low- or non-fat dairy, while restricting sweets, saturated fats, and especially sodium [24]. A substantial body of evidence supports the cardioprotective effects of the Mediterranean and DASH dietary patterns [25,26]. Additionally, current studies are investigating the potential of these dietary patterns as a preventive measure for cognitive disorders, including AD [27].

**Figure 1:**
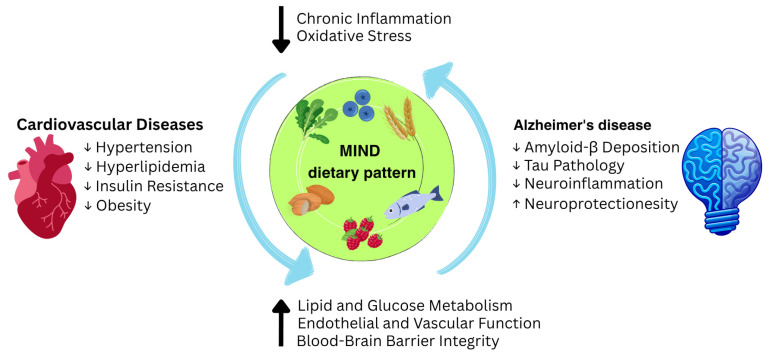
The MIND Dietary Pattern as a Central Preventative Strategy for Cardiovascular Diseases and Alzheimer’s Disease.

In 2015, Morris and colleagues proposed the Mediterranean–DASH Intervention for Neurodegenerative Delay (MIND) dietary pattern, a hybrid of the Mediterranean and DASH dietary patterns specifically designed to protect cognitive health by emphasizing consumption of the foods and nutrients that protect against cognitive decline and incident dementia [27]. The MIND dietary pattern emphasizes ten brain-protective foods, including green leafy vegetables, other vegetables, berries, nuts, beans, whole grains, fish, poultry, olive oil, and wine, while restricting the intake of cheese, red meat and its products, fast foods or fried foods, pastries, sweets, butter, and margarine [27]. Although the MIND dietary pattern shares key components with the Mediterranean and DASH dietary patterns, it uniquely highlights berries and leafy greens, with serving sizes based on diet–dementia study findings [28]. Accumulating evidence suggests a positive association between adherence to the MIND dietary pattern and improved cognitive function through different mechanisms, including inflammatory pathways [29,30]. This protective effect may be attributed to components of the MIND dietary pattern, which include antioxidants and brain-healthy nutrients such as vitamin E, folate, polyphenols, flavonoids, carotenoids, fiber, monounsaturated fatty acids (MUFAs), and omega-3 fatty acids, which may inhibit Aβ deposition and contribute to the prevention of AD, either directly or indirectly by affecting CVD risk factors [31,32,33,34,35,36,37,38]. Additionally, adherence to the MIND dietary pattern has also been associated with reduced CVD risk factors, supporting its role in promoting both cardiovascular and cognitive health [39]. Therefore, the MIND dietary pattern might be a key lifestyle strategy for reducing the risk of both CVDs and AD simultaneously (Figure 1).

Although previous evidence suggests the MIND dietary pattern as a potential prevention and management strategy for both conditions, research remains limited in examining the overlapping mechanisms between CVD risk factors and AD, as well as in evaluating clinical evidence specifically related to AD. Therefore, the current literature/narrative review aims to synthesize the published literature and highlight the underlying mechanisms from preclinical to clinical studies to elucidate the relationship between CVD risk factors, AD, and the role of the MIND dietary pattern in these conditions. To identify relevant studies a broad literature search in databases including PubMed, Scopus, and Web of Science from inception until April 2025 was used. Keywords included combinations of ‘MIND diet’, ‘cardiovascular disease’, ‘Alzheimer’s disease’, ‘oxidative stress’, and ‘cognition’. Due to the narrative nature of the review, no formal inclusion/exclusion criteria were defined, and selection was based on relevance and quality of evidence.

## 2. CVD Risk Factors and AD

### 2.1. Hypertension

Among the CVD risk factors, HTN has the most robust causal evidence [40]. Chronic uncontrolled HTN can lead to hypertensive heart disease, which refers to a range of abnormalities in the left ventricle (LV), left atrium, and coronary arteries [41]. Common complications of hypertensive heart disease include diastolic heart failure, systolic heart failure, or a combination of both [41].

Longitudinal studies found that blood pressure levels are elevated long before (even decades before) the onset of AD [42,43]. Additionally, these studies suggest that midlife and late-life HTN are associated with increases in pathological changes associated with AD [44]. For example, a study by Lennon et al. revealed that midlife HTN, stage 1 (systolic blood pressure > 140 mmHg) and stage 2 (systolic blood pressure > 160 mmHg), are associated with an 18% and 25% increase in the risk of AD, respectively [44]. The exact mechanisms for this co-occurrence are still unclear and are being investigated. It has been suggested, however, that HTN may result in altered cerebral autoregulatory mechanisms by damaging the cerebral vasculature endothelium. This can ultimately lead to cerebral hypoperfusion and, subsequently, cognitive deficits [45]. Furthermore, studies have reported an association between high blood pressure and a disrupted BBB, which is an early sign of AD. HTN-induced oxidative stress in cerebral vessels leads to increased activity of matrix metalloproteinases, which degrade tight junction proteins of the BBB [46]. When glial cells release these enzymes, they have the potential to harm both myelin and synapses [47]. Myelin impairment has been identified as early indicator of AD pathology, occurring before the onset of typical pathological changes such as formation of NFTs [48]. However, it remains unclear whether myelin damage directly triggers amyloidogenesis [48].

### 2.2. Dyslipidemia

Studies have suggested that serum lipids could accumulate in the heart, triggering oxidative stress and inflammatory cardiac fibrosis, reducing autophagy and microvascular density, and altering the mitochondrial function of cardiomyocytes [49]. These changes make the myocardium more susceptible to damage, potentially resulting in cardiac dysfunction and electrophysiological alterations. Therefore, reducing serum lipid levels may help to reverse early ventricular dysfunction and offer cardioprotective effects [49].

Moreover, several studies, from laboratory to clinical investigations, have explored the relationship between lipids and/or lipid-lowering treatments and AD, and they have indicated a positive association between dyslipidemia and the risk of AD [50,51,52,53]. These results are supported by genetic linkages and observational studies, which have identified multiple distinct genes involved in cholesterol metabolism or transport as susceptibility genes for AD. These include apolipoprotein E (APOE), apolipoprotein J (APOJ, also known as CLU), ATP-binding cassette subfamily A member 7 (ABCA7), and the sortilin-related receptor (SORL1) [50,54,55,56,57,58]. Cell biology studies provide additional evidence for the key role of lipid raft cholesterol in regulating the processing of Aβ precursor protein by β-secretase and γ-secretase, which results in altered Aβ production [50,59,60]. Moreover, a substantial body of population-based observational studies has shown that 3-hydroxy-3-methylglutaryl coenzyme A reductase inhibitors, known as statins, may protect against the risk of AD and dementia [61,62,63,64]. However, the majority of randomized controlled trials (RCTs) have found no beneficial effects of statins on cognitive decline or dementia risk [65,66,67,68,69]. These null results can be explained by the relatively short period of these trials and the inclusion of patients with advanced AD [50].

### 2.3. Diabetes Mellitus

CVDs are the leading cause of morbidity and mortality among individuals with DM, the majority of whom (approximately 90–95%) have type 2 diabetes (T2DM) [70,71]. T2DM can be involved in the development of CVD directly or indirectly by its role in the development of other CVD risk factors such as obesity, dyslipidemia, or HTN [72]. T2DM may play a direct role in the development of cardiomyopathy, beyond its known association with coronary atherosclerosis and HTN [72]. This form of diabetic cardiomyopathy has been observed in numerous noninvasive studies, highlighting structural and functional changes in the LV of adults with diabetes [72]. Notably, people with diabetes tend to exhibit greater cardiac mass, particularly LV mass, compared to people without diabetes [73,74]. This increased cardiac mass, or hypertrophy, may be associated with the elevated release of adipocyte-derived cytokines, such as leptin and resistin, which exert hypertrophic effects on cardiomyocytes [75,76]. Moreover, T2DM has also been linked to a higher risk of myocardial infarction, which might be attributed to increased coagulability in T2DM [72,77].

An extensive body of epidemiological studies suggests that people with T2DM are at a higher risk of developing AD [78,79]. Several mechanisms have been proposed for this relationship, including the role of insulin resistance in exacerbating Aβ and tau pathologies. Insulin resistance, a key characteristic of T2DM, can enhance the production and release of Aβ by reducing its breakdown via the insulin-degrading enzyme [80,81,82,83]. In addition, insulin resistance disrupts the PI3K/AKT/GSK-3β signaling pathway, resulting in the formation of hyperphosphorylated tau [83,84]. This condition also causes synapse loss, impairs autophagy, and increases neuronal apoptosis [83]. These changes may initiate a chain reaction that leads to the abnormal buildup of Aβ and tau, ultimately contributing to the development of AD pathology. Overall, these findings suggest that DM increases susceptibility to AD [85].

### 2.4. Obesity

Obesity may affect CVD through its association with other known risk factors such as insulin resistance, HTN, metabolic syndrome, T2DM, and atherosclerosis [86]. These conditions are promoted by visceral white adipocyte tissue dysfunction through chronically elevated pro-inflammatory adipokines (compared to people without obesity), oxidative stress, renin–angiotensin–aldosterone system activation, and an adverse gut microbiome. Inflammation and oxidative stress in adipose tissue lead to a decrease in the production of adiponectin, and elevated secretion of resistin, leptin, and pro-inflammatory adipokines and cytokines. These changes contribute to increased arterial stiffness and reduced vascular relaxation and ultimately lead to cardiac diastolic dysfunction [17]. Further, activation of the renin–angiotensin–aldosterone system in obesity, which plays an important role in the hemostasis of the cardiovascular system, stimulates inflammation and structural remodeling under pathophysiological conditions, thus inducing cardiac and vascular injury [17,86,87].

Epidemiological studies have reported a higher risk of AD in people who are overweight or obese [88,89]. There are several potential explanatory mechanisms for this relationship, with an emphasis on the positive correlation between high-fat-diet-induced obesity and Aβ accumulation [90,91]. Studies have reported increased amyloid precursor proteins (APPs), known as the precursor molecule that generates Aβ through its proteolysis in adipose tissue and Aβ in the plasma of people with obesity compared to those without obesity [92,93]. The increase in Aβ plasma levels in middle-aged people with obesity may be due to enhanced adipocyte APP gene expression [93]. Chronically elevated Aβ plasma levels may lead to elevated transportation of Aβ into the human brain through Receptors for Advanced Glycation End (RAGE) products and ultimately contribute to the development of AD [90]. Further possible mechanisms for the relationship between obesity and AD include the production of proinflammatory cytokines and adipokines, excess oxidative stress generation and mitochondrial dysfunction, insulin resistance, loss of BBB integrity, and ceramide production [90].

### 2.5. Smoking

Smoking is perhaps the best known risk factor for CVDs, with substantive evidence showing that it contributes to cardiovascular morbidity and mortality [94,95]. The primary processes involved in smoking-induced CVDs―particularly atherogenesis―include endothelial dysfunction and damage, increases in and oxidation of proatherogenic lipids, reductions in high-density lipoprotein (HDL), heightened inflammatory status, and a shift in the circulatory system toward a procoagulant state [96]. In brief, through a reduction in NO bioavailability, smoking can induce vascular dysfunction, which leads to increased expression of adhesion molecules and ultimately endothelial dysfunction [96,97]. The smoking-induced heightened adhesion of platelets and macrophages creates a procoagulant and inflammatory environment [96,98]. Following transendothelial migration and activation, macrophages absorb oxidized lipoproteins produced through oxidative modifications, transforming into foam cells, which play a crucial role in lipid deposition and plaque formation within the arterial walls [96].

Smoking is also a well-established risk factor for AD, and previous studies have revealed that smokers have a higher risk of cognitive impairment and AD compared to non-smokers [99]. Based on in vitro, animal, and human studies, chronic exposure to cigarette smoke and nicotine is associated with oxidative stress [100,101]. Smoking is closely associated with cerebral oxidative stress, which promotes β-secretase cleavage of APPs and contributes to abnormal tau phosphorylation [101,102,103,104]. Therefore, smoking-induced oxidative stress may directly upregulate the amyloidogenic pathway, leading to Aβ oligomer production and extracellular fibrillar Aβ aggregation [101]. Oxidative stress also causes abnormal tau phosphorylation, a fundamental process underlying neurofibrillary tangle pathology [101].

### 2.6. Physical Inactivity

Globally, 7.6% of CVD deaths are attributable to physical inactivity, defined as not obtaining at least 150 min of moderate-intensity or 75 min of vigorous-intensity physical activity per week, or an equivalent mix of both [105]. Physical inactivity may lead to the impairment of glucose homeostasis and lipid metabolism through a reduction in muscle glucose transporter type 4 content and insulin-stimulated glucose uptake [106]. Physical inactivity may also decrease the activity of lipoprotein lipase, which leads to impairment in triglyceride (TG) and high-density lipoprotein cholesterol (HDL-C) metabolism and ultimately results in the development of CVDs [106,107].

Estimates suggest that approximately 13% of all AD cases worldwide may be attributable to physical inactivity [108]. Moreover, studies have revealed that people with high levels of sedentary behavior, commonly defined as activities involving an energy expenditure of ≤1.5 metabolic equivalents (METs) while sitting, reclining, or lying down, are at a higher risk of AD development compared to those with lower levels of sedentary time [109,110,111]. This association may be explained by sedentary behavior’s impact on neuroinflammation, potentially accelerating the accumulation of Aβ and tau protein [112,113]. The accumulation of Aβ plaques in AD causes the activation of microglia, a category of mononuclear phagocytes/macrophages of hematopoietic origin, found in the central nervous system, resulting in synaptic phagocytosis and therefore, neurodegeneration. Animal and human studies have shown that regular physical activity has the potential to inhibit microglial activation and improve AD pathogenesis by reducing the expression of inflammatory cytokines (e.g., Interleukin-1β and tumor necrosis factor-α) [114,115,116]. Regular physical activity and exercise may also improve endothelial function by increasing the frictional forces, such as shear stress, exerted on the endothelium of the vascular walls by blood flow [114]. Endothelial shear stress triggers the production of vasodilatory substances, including NO, and enhances the expression and activation of endothelial NO synthase, thereby facilitating revascularization [114,117]. These mechanisms ultimately protect the integrity of the BBB [118].

## 3. MIND Dietary Pattern and CVD Risk Factors

### 3.1. MIND Dietary Pattern and Hypertension

Although previously reported results from studies of the relationship between adherence to the MIND dietary pattern and HTN risk have not been conclusive, some observational studies have suggested a significantly lower prevalence of HTN in participants with higher adherence to the MIND dietary pattern compared to those with lower adherence [119,120,121]. The beneficial effect of the MIND dietary pattern on blood pressure was confirmed in one RCT by Yau et al. (Table 2) [122].

The MIND dietary pattern recommends high consumption of fruits and vegetables, which are associated with a high intake of potassium, magnesium, and fiber. These components are associated with lower blood pressure in observational and interventional studies [127,128]. Additionally, this dietary pattern limits the intake of highly processed foods that contain high amounts of sodium. Although the effects of sodium on blood pressure vary among individuals, salt-sensitive individuals may experience HTN due to excessive dietary salt intake (Table 3) [129]. As compared to people with usual salt-sensitivity, individuals who have salt-sensitivity that results in elevations in blood pressure have a dysfunctional renin-angiotensin system, meaning there is reduced renin stimulation during salt depletion, and the system fails to adequately suppress renin in response to high salt intake, thereby worsening the adverse effects of salt on blood pressure [130,131]. Moreover, evidence suggests that adequate potassium intake—which is promoted by the MIND dietary pattern due to its emphasis on fruits and vegetables—is desirable to achieve lower blood pressure [132]. Several explanations have been proposed for this effect of potassium, including its role in reducing vascular smooth muscle contraction by altering membrane potential or restoring endothelium-dependent vasorelaxation [133,134]. However, due to the U-shaped associations between high serum potassium levels and the risks of adverse outcomes in observational studies, excessive potassium supplementation should be avoided [132]. Moreover, the preponderance of evidence supports a protective effect of magnesium against HTN [37,135,136]. Magnesium—which is promoted by the MIND dietary pattern due to its emphasis on green leafy vegetables, whole grains, legumes, and seeds—acts as a calcium channel blocker [37,135,136]. It prevents sodium from attaching to vascular smooth muscle cells, increases the production of the vasodilating prostaglandin E, and binds potassium cooperatively. Additionally, magnesium boosts NO levels, improves endothelial function, promotes vasodilation, and lowers blood pressure [37].

### 3.2. MIND Dietary Pattern and Dyslipidemia

Overall, previous investigations have shown beneficial effects of the MIND dietary pattern on lipid biomarkers [39]. Observational studies have reported a positive association between the MIND dietary pattern and HDL-C, and a negative association with total cholesterol (TC)/HDL-C ratio [121,137,138]. However, some differences have been observed across studies concerning the association between the MIND dietary pattern and TG, which have been attributed to overall high meat and margarine consumption within the populations studied, items that are limited in the MIND dietary pattern [39,121,138]. Furthermore, two clinical trials explored the potential effects of the MIND dietary pattern and confirmed its beneficial role in dyslipidemia [122,123]. The RCTs reported reductions in TG, TC, and low-density lipoprotein cholesterol (LDL-C) in participants who adhered to the MIND dietary pattern compared to the control group (Table 2) [122,123]. Additionally, one of the RCTs reported a significant increase in HDL-C in the MIND diet group compared to the control group [123], whereas the other found no significant effect, potentially due to the relatively shorter duration of the study (4 weeks) compared to the other (12 weeks) (Table 2) [122].

The beneficial effects of the MIND dietary pattern on lipid biomarkers can be explained by the dietary components it promotes and limits. The MIND dietary pattern is characterized by high amounts of vegetables, berries, nuts, beans, and whole grains, resulting in a high fiber intake. Accumulating evidence supports the lipid-lowering effects of fiber (Table 3) [31,32,33].

Several mechanisms have been proposed to explain how dietary fiber reduces serum lipids: for example, fiber binds to bile acids, increases viscosity, and creates bulk in the small intestine, which suppresses the absorption of glucose and lipids [33]. Additionally, dietary fiber promotes the production of short-chain fatty acids (SCFAs), which have a lipid-lowering effect, and modulates genes associated with lipid metabolism [33]. Moreover, by promoting the consumption of olive oil, fish, and nuts, the MIND dietary pattern provides appropriate proportions of polyunsaturated fatty acids (PUFAs) and MUFAs. Evidence has suggested that the consumption of MUFAs, which can be found in vegetable oils such as olive and canola, is associated with increased HDL-C levels and decreased LDL-C and TG [139,140,141]. Furthermore, the omega-3 PUFA family, which can be found in flaxseed, walnuts, chia seeds, soybeans, hemp seeds, algae, mackerel, herring, and salmon, contributes to the inhibition of the endogenous synthesis and esterification of cholesterol, an increase in cholesterol excretion in the bile, and bile salt synthesis [142]. Additionally, omega-3 PUFAs contribute to lowering plasma TGs by lowering very low-density lipoprotein (VLDL) synthesis in the liver [142]. Other potential mechanisms for the benefits of the MIND dietary pattern on hyperlipidemia include the antioxidant content of the MIND dietary pattern, such as polyphenols and flavonoids, which can inhibit the synthesis of endogenous cholesterol and decrease the risk of CVDs [142].

### 3.3. MIND Dietary Pattern and Diabetes Mellitus

Despite some inconsistency among the results of observational studies that have investigated the relationship between the MIND dietary pattern and T2DM, the majority have reported a negative association between adherence to the MIND dietary pattern and the risk of T2DM and glucose levels [121,137,138,143,144]. Notably, these findings are in line with the results of the two available RCTs by Yau et al. and Gholami et al., which reported reductions in glucose levels in participants assigned to the MIND diet intervention compared to the control groups (Table 2) [122,123].

The MIND dietary pattern can contribute to the prevention of T2DM through several mechanisms, including reductions in inflammation and insulin resistance. As inflammation is a key mechanism in the pathogenesis of CVD risk factors and especially T2DM, the benefits of the MIND dietary pattern on T2DM can be attributed to its rich antioxidant and anti-inflammatory compounds [36]. Dietary antioxidants, including vitamins A, E, and C, plant polyphenols, carotenoids, flavonoids, glutathione, alpha-lipoic acid, and polyamines, are known for their protective effects against T2DM (Table 3). Studies have shown that antioxidant treatments, including dietary antioxidants and supplements, protect beta-cells from oxidative stress-induced apoptosis, help maintain beta-cell function, and reduce complications associated with T2DM [145,146]. Furthermore, dietary fiber may improve insulin resistance through gut microbiome-derived SCFAs, while PUFAs do so by the suppression of TLR2/4 signaling and activation of the peroxisome proliferator-activated receptor [147].

### 3.4. MIND Dietary Pattern and Obesity

Previous studies have revealed a significant beneficial effect of the MIND dietary pattern on obesity and anthropometric indicators, including reduced waist circumference, body mass index (BMI), and waist-to-hip ratio (WHR) [121,122,124,125,137]. In particular, clinical trials have reported reductions in waist circumference, BMI, WHR, and body weight for MIND dietary pattern groups in comparison to control groups [122,123,124,125]. However, the effects of the MIND dietary pattern on body fat percentage were inconsistent, potentially due to the heterogeneity in body fat at baseline [39].

The favorable effects of the MIND dietary pattern on anthropometric indices can be attributed to several mechanisms, including the restriction of high-calorie foods and emphasis on the increased consumption of fiber and antioxidants. Limiting high-calorie foods, such as highly processed foods or sweets, can result in a lower energy intake and, ultimately, a lower prevalence of obesity (Table 3). The protective mechanism of fiber against obesity includes decreased absorption of macronutrients and enhanced satiety [34]. Additionally, beneficial alterations in gut microbiota and SCFA production may underpin the protective effects of high-fiber diets against obesity and may suggest their potential role in the treatment of obesity [34,35]. Moreover, recent studies highlight the role of oxidative stress in the development of obesity by stimulating the deposition of adipose tissue, including preadipocyte proliferation, and adipocyte differentiation and growth [148,149]. Therefore, a high intake of antioxidants in the MIND dietary pattern may also contribute to the prevention of obesity.

## 4. MIND Dietary Pattern and AD

**Figure 2:**
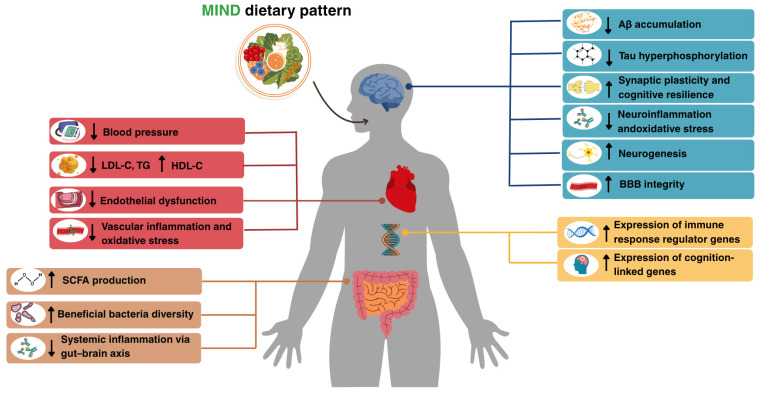
Proposed Biological Mechanisms Linking the MIND Diet to Cardiovascular Disease and Alzheimer’s Disease Pathology. Abbreviations: Aβ, amyloid-β; BBB, blood–brain barrier; HDL-C, high-density lipoprotein cholesterol; LDL-C, low-density lipoprotein cholesterol; SCFA, short-chain fatty acids; TG, triglycerides; MIND, Mediterranean–DASH Intervention for Neurodegenerative Delay.

The impact of the MIND dietary pattern on AD goes beyond the relationship between CVD risk factors and AD, involving neuroprotective antioxidant and anti-inflammatory pathways, transcriptomic changes linked to cognitive resilience, and gut microbiota modulation (Figure 2).

One of the central mechanisms for the association between the MIND dietary pattern and AD involves the high content of antioxidants, such as vitamin E, vitamin C, carotenoids (e.g., lutein, beta-carotene), and polyphenols found in green leafy vegetables, berries, nuts, and olive oil (Table 3). These components play a crucial role in neutralizing reactive oxygen species (ROS), which are elevated in patients with AD and contribute to Aβ plaque formation by upregulating the amyloidogenic processing of APP, mainly through the increased activity of enzymes like β-site APP-cleaving enzyme 1 (BACE1) and γ-secretase, enzymes involved in the production of Aβ peptides [150,151]. Additionally, antioxidants hold the potential to reduce oxidative stress, which can trigger the phosphorylation of tau, reducing its ability to bind to microtubules, which leads to their destabilization and ultimately contributes to the development of NFTs [18].

The MIND dietary pattern is rich in anti-inflammatory components such as omega-3 fatty acids, which downregulate microglial activation and pro-inflammatory signaling pathways such as nuclear factor kappa B (NF-κB) [152,153]. Downregulation of NF-κB is associated with decreased BACE1 expression and ultimately lower Aβ production [154]. Moreover, folate, found abundantly in green leafy vegetables and legumes—foods emphasized in the MIND dietary pattern—contributes to a reduction in the plasma homocysteine level, which is elevated in AD [155,156].

Recent studies suggest that soy isoflavones, especially genistein, could have a positive role in AD through antioxidant, anti-inflammatory, and estrogenic effects. In vitro and in vivo studies have shown that genistein enhances antioxidant gene expression, supports neuronal survival during apoptosis, and reduces amyloid-β accumulation by modulating BACE1 activity and oxidative stress pathways [157,158,159,160]. Additionally, a recent RCT by Viña et al. found a significant reduction in amyloid-β accumulation in the anterior cingulate gyrus and improvements in cognitive performance after 12 months of genistein supplementation in patients with prodromal AD [161]. Furthermore, a systematic review and meta-analysis of RCTs demonstrated that genistein supplementation could significantly reduce several CVD risk factors, including TC, LDL-C, systolic and diastolic blood pressure, fasting blood glucose, fasting insulin, HOMA-IR, and homocysteine levels, which could consequently reduce the risk of AD [162].

Emerging evidence suggests a role for the MIND dietary pattern in promoting cognitive resilience through transcriptomic changes linked to brain health [38]. A cross-sectional study by Li et al. explored the connection between the MIND dietary pattern and AD by using RNA sequencing data from post-mortem prefrontal cortex tissue and annual cognitive evaluations from 1204 participants. Their results revealed that the adherence to the MIND dietary pattern, compared to those with lower adherence, was correlated with a specific brain transcriptomic profile, consisting of 50 genes, which were associated with slower cognitive decline and lower odds of dementia [38]. For example, the immune response regulator (TCIM) gene, which showed the strongest positive correlation with the MIND diet score, encodes a transcriptional and immune-response regulator that activates the wingless-related integration site/beta-catenin (Wnt/β-catenin) signaling pathway [163]. This pathway plays a role in neuronal development and survival, and it suppresses APPs by downregulating BACE1. Additionally, evidence suggests a negative association between Wnt/β-catenin pathway activation and tau phosphorylation, mediated through the regulation of glycogen synthase kinase-3β (GSK-3β), a key enzyme involved in tau hyperphosphorylation [164].

Moreover, clinical studies on AD patients have shown that diets rich in antioxidant and anti-inflammatory nutrients promote the growth of beneficial gut microbiota, which are often diminished in AD [165]. One such example is the Bifidobacterium, which plays a role in maintaining a balanced microbial state (eubiosis) [165]. When this balance is disrupted (dysbiosis), it can lead to the production of bacterial toxins that contribute to brain amyloidogenesis [166,167]. Specifically, dysbiosis may result in the release of neurotoxic metabolites such as lipopolysaccharides (LPSs), which can cross the BBB, trigger neuroinflammation, and upregulate amyloidogenic enzymes like BACE1 and γ-secretase, ultimately leading to increased Aβ production [168].

## 5. Discussion

There is a substantial body of evidence that supports the connections between CVDs and AD [10,11,12]. Additionally, the pathologies of these conditions start decades before the development of clinical events such as myocardial infarction, heart failure, cognitive impairment, or death. Therefore, there is a prolonged window of opportunity to implement prevention strategies in order to reduce the risk of both CVDs and AD simultaneously.

Due to the connections between CVDs and AD, the management of CVD risk factors, including HTN, dyslipidemia, DM, obesity, smoking, or physical inactivity, might be critical for the prevention of both CVDs and AD. Healthful dietary patterns, in particular, hold the potential to contribute to the prevention of most CVD risk factors, and ultimately, AD [30,39,169]. Specifically, healthy plant-based dietary patterns including Mediterranean, DASH, or the MIND dietary pattern are associated with reduced risk of both CVD risk factors and AD [39,170]. Convincing evidence from both observational studies and clinical trials has suggested a beneficial role for the MIND dietary pattern in CVD prevention [39,122,123]. In agreement with the current narrative review, a systematic review and meta-analysis by Akbar et al. found that the MIND dietary pattern was significantly associated with reduced CVD risk, including anthropometric measures, blood pressure, glycemic control, lipid profiles, and inflammation [39]. Furthermore, evidence suggests that the strongest positive associations between dietary pattern and cognitive function were observed for the MIND dietary pattern [171].

While experimental human studies remain limited, recent systematic review articles explored animal and epidemiological studies and revealed a strong negative association between adherence to the MIND dietary pattern and dementia [30,169]. However, the three available RCTs to date have reported conflicting findings (Table 2) [30,146,147,172]. For example, an RCT conducted in the U.S. by Barnes and colleagues found no effect of a 3-year MIND diet intervention on cognitive function in older adults who were overweight [126]. In contrast, a relatively small Iranian trial by Arjmand et al. involving middle-aged women with obesity demonstrated short-term beneficial cognitive effects of the MIND diet intervention [172]. After a 3-month intervention, participants in the MIND diet group demonstrated improvements in cognitive function compared to the control group. The observed inconsistencies among existing RCTs may stem from methodological limitations, including short intervention durations (e.g., 12 weeks in Arjmand et al. [124] and Elsayed et al. [125] versus 3 years in Barnes et al. [126]), relatively small sample sizes (*n* = 40–68 in the Arjmand et al. and Elsayed et al. studies), lack of blinding, heterogeneity in baseline cognitive function, differences in control groups, and the inclusion of participants with a family history of cognitive disorders (Barnes et al. study). Additionally, the outcome measures varied considerably—from subjective neuropsychological assessments such as working memory, attention, and verbal fluency to objective neuroimaging outcomes, including hippocampal volume and white matter hyperintensities. Future studies should prioritize standardized protocols, longer durations, and consistent, validated tools for the assessment of cognitive health (Table 2).

**Figure 3:**
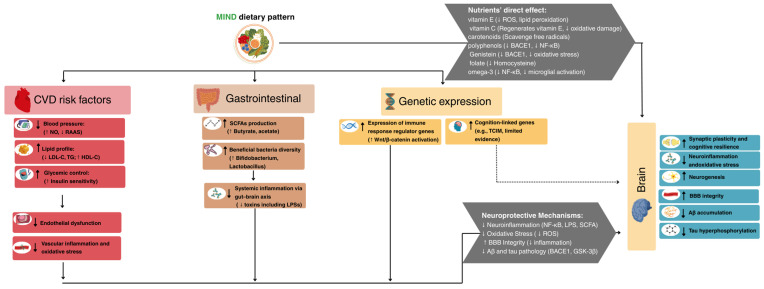
Mechanistic Pathways Linking the MIND Dietary Pattern to Alzheimer’s disease. Abbreviations: Aβ: amyloid-β; BACE1: β-site amyloid precursor protein-cleaving enzyme 1; BBB: blood–brain barrier; CVD: cardiovascular disease; GSK-3β: glycogen synthase kinase 3 beta; HDL-C: high-density lipoprotein cholesterol; LDL-C: low-density lipoprotein cholesterol; LPSs: lipopolysaccharides; MIND: Mediterranean–DASH Intervention for Neurodegenerative Delay; NF-κB: nuclear factor kappa-light-chain-enhancer of activated B cells; NO: nitric oxide; RAAS: renin–angiotensin–aldosterone system; ROS: reactive oxygen species; SCFAs: short-chain fatty acids; TG: triglycerides; TCIM: transcriptional and immune response modulator gene.

The beneficial impacts of the MIND dietary pattern for CVDs and AD can be explained by the role of the MIND dietary pattern in reducing CVD risk factors, neuroinflammation and oxidative stress, transcriptomic changes linked to cognitive resilience, and gut microbiota modulation (Figure 3).

The MIND dietary pattern is rich in antioxidants, fiber, MUFAs, omega-3 fatty acids, polyphenols, and flavonoids. These components promote vascular health, improved lipid profiles, enhanced glucose metabolism, and better anthropometric indices through various mechanisms, particularly through decreased inflammation (Table 3) [31,32,33,34,35,36,37]. These improvements can ultimately result in improved cerebral blood flow, decreased neuroinflammation and enhanced neurogenesis and can promote neuroplasticity, which collectively affect Aβ production and metabolism [170]. It is also worth noting that the beneficial impacts of the MIND dietary pattern on cognitive and cardiovascular health may extend beyond individual foods. Rather than isolated foods and nutrients, the synergistic effects of the overall dietary pattern may better predict health outcomes.

While our narrative approach has provided a broad synthesis of mechanisms, it may have overlooked other nuanced interactions due to the non-systematic literature selection and potential selection bias. Additionally, most available studies emphasized positive associations, which may have limited our ability to reflect mixed, null, or negative findings. Therefore, prospective studies employing systematic methodologies―including systematic reviews, meta-analyses of intervention studies, and carefully controlled prospective cohort designs―are needed to further validate these mechanisms and findings. Additionally, future research should explore currently understudied areas in greater depth—particularly the influence of the MIND dietary pattern and other nutritional interventions on the transcriptomic changes associated with cognitive resilience, as well as the temporal dynamics, dose-dependent effects, and robust human clinical evidence required to clarify these relationships. Lastly, the current evidence has focused mainly on exploring individual nutrients and foods rather than the MIND dietary pattern as a whole. This underscores the need for future research to investigate the complex interactions, synergistic effects, and both acute and chronic impacts of dietary patterns as integrated systems.

## 6. Conclusions

There is a substantial body of evidence to support the connections between CVD risk factors and AD. The MIND dietary pattern has been suggested to play a beneficial role in these conditions through multiple distinct mechanisms, including reductions in oxidative stress and inflammation, modulation of the gene expression associated with cognitive resilience, improvements in vascular and metabolic health, preservation of BBB integrity, inhibition of amyloid-β and tau pathology, and gut microbiota modulation. Based primarily on preclinical studies, the MIND dietary pattern may inhibit Aβ deposition and contribute to the prevention of AD, either directly or indirectly by affecting CVD risk factors. However, only three RCTs have investigated the effects of the MIND dietary pattern on cognitive health, with inconsistent findings. Therefore, further long-term RCTs are required to elucidate the potential role of the MIND dietary pattern in preventing and possibly in managing the symptoms of AD. 

## Figures and Tables

**Table 1:** Overview of Cardiovascular Disease Risk Factors and Their Proposed Mechanisms in Alzheimer’s Disease Pathogenesis.

CVD Risk Factor	CVD-Related Mechanisms	AD-Related Mechanisms	Evidence Type	Strength of Evidence	Reversibility
**HTN**	Endothelial dysfunction, cerebral hypoperfusion, BBB disruption	Elevated Aβ and tau pathology; damage to myelin and synapses	Human, Animal	Strong	Partially reversible with blood pressure control
**Dyslipidemia**	Lipid accumulation, oxidative stress, mitochondrial dysfunction	Alters Aβ production; associated with cholesterol metabolism genes (e.g., APOE, SORL1)	Human, Animal, Genetic	Moderate to Strong	Partially reversible with statins/diet
**DM**	Insulin resistance, cardiac remodeling, increased inflammation	Enhances Aβ accumulation (via reduced insulin-degrading enzyme activity); promotes tau hyperphosphorylation	Human, Animal	Strong	Partially reversible with glycemic control
**Obesity**	Adipokine dysregulation, oxidative stress, RAAS activation	Increased APP and Aβ in adipose tissue; elevated plasma Aβ; BBB disruption; mitochondrial dysfunction	Human, Animal	Moderate	Partially reversible with weight loss
**Smoking**	Endothelial damage, inflammation, oxidative stress	Increases Aβ aggregation and tau pathology via oxidative stress	Human, Animal	Strong	Largely irreversible, but further damage preventable
**Physical Inactivity**	Impaired glucose/lipid metabolism, endothelial dysfunction	Increase neuroinflammation, accelerating the accumulation of Aβ and tau protein; reduces BBB integrity	Human, Animal	Moderate	Reversible with regular physical activity

Abbreviations: Aβ, amyloid-β; APP, amyloid precursor protein; APOE, apolipoprotein E; BBB, blood–brain barrier; DM, diabetes mellitus; HTN, hypertension; RAAS, renin–angiotensin–aldosterone system; SORL1, sortilin-related receptor 1.

**Table 2:** Summary of Clinical Trials on the MIND Diet and Cognitive/Cardiometabolic Outcomes.

Study	Country	Sample Size	Population	Duration	Outcomes Measured	Key Findings
**Yau et al. (2022) [122]**	China	78	Older Chinese adults	4 weeks	BP, glucose, HDL-C, mental health	↓ BP, ↓ glucose, ↑ HDL-C, improved mental well-being
**Gholami et al. (2024) [123]**	Iran	84	Adults with metabolic syndrome	12 weeks	Weight, BMI, WC, SBP, DBP, FBS, HDL-C, TG	↓ BMI, WC, BP, FBS, TG; ↑ HDL-C.
**Arjmand et al. (2022) [124]**	Iran	40	Middle-aged overweight/obese women	12 weeks	Cognitive performance, brain MRI (IFG surface area), BMI, WHR, body weight	↑ working memory, attention, verbal memory; ↑ IFG surface area; ↓ BMI, WHR, weight
**Elsayed et al. (2022) [125]**	Egypt	68	Postmenopausal women with hormone deficiency	12 weeks	Cognitive & functional level, sex hormone markers	↑ cognition and functionality with MIND + aerobic exercise vs MIND alone
**Barnes et al. (2023) [126]**	United States	604	Older overweight adults	3 years	Global cognition, MRI brain markers (WMH, hippocampal volume)	No significant difference in cognition or MRI outcomes vs control; both groups improved slightly

Abbreviations: BMI, body mass index; BP, blood pressure; DBP, diastolic blood pressure; FBS, fasting blood sugar; HDL-C, high-density lipoprotein cholesterol; IFG, inferior frontal gyrus; MIND, Mediterranean–DASH Intervention for Neurodegenerative Delay; MRI, magnetic resonance imaging; SBP, systolic blood pressure; TG, triglycerides; WC, waist circumference; WMH, white matter hyperintensities; WHR, waist-to-hip ratio.

**Table 3:** Nutritional Components of the MIND Dietary Pattern and Their Proposed Effects on CVDs and AD.

Dietary Component	Key Nutrients	Proposed Effects on CVD	Proposed Effects on AD
**Green leafy vegetables**	Folate, potassium, magnesium, fiber	Lower BP via vasodilation and endothelial support	Reduces oxidative stress, lowers homocysteine levels, supports cognitive resilience
**Berries**	Polyphenols, flavonoids	Anti-inflammatory, improves lipid profile	Protects against Aβ accumulation and oxidative damage
**Nuts**	MUFAs, vitamin E, polyphenols	Improves HDL-C, lowers LDL-C, reduces inflammation	Enhances synaptic function, reduces tau pathology
**Whole grains**	Fiber, B vitamins, antioxidants	Lowers cholesterol, improves glycemic control	Produces SCFAs, reduces inflammation, improves gut-brain axis
**Fish**	Omega-3 PUFAs	Reduces TGs and inflammation	Downregulates NF-κB, lowers BACE1 activity, reduces Aβ and tau production
**Olive oil**	MUFAs, polyphenols, vitamin E	Improves lipid profile, lowers BP, reduces oxidative stress	Has antioxidant and anti-amyloidogenic effects
**Beans and legumes**	Folate, fiber, magnesium	Supports lipid and glucose metabolism	Reduces oxidative stress and inflammation
**Restricted items (e.g., red/processed meats, sweets, butter)**	Saturated fats, sodium, refined sugars	Reduces risk of obesity, dyslipidemia, HTN	Promotes Aβ accumulation and cognitive decline

Abbreviations: Aβ, amyloid-β; AD, Alzheimer’s disease; BACE1, β-site amyloid precursor protein-cleaving enzyme 1; BP, blood pressure; CVD, cardiovascular disease; HDL-C, high-density lipoprotein cholesterol; HTN, hypertension; LDL-C, low-density lipoprotein cholesterol; MUFAs, monounsaturated fatty acids; NF-κB, nuclear factor kappa-light-chain-enhancer of activated B cells; PUFAs, polyunsaturated fatty acids; SCFAs, short-chain fatty acids; TGs, triglycerides.

## Abbreviations

The following abbreviations are used in this manuscript:

Aβ	Amyloid-beta
AD	Alzheimer’s Disease
APOE	Apolipoprotein E
APOJ	Apolipoprotein J
APP	Amyloid Precursor Protein
BBB	Blood–Brain Barrier
BMI	Body Mass Index
CVDs	Cardiovascular Diseases
DASH	Dietary Approaches to Stop Hypertension
DM	Diabetes Mellitus
GSK-3β	glycogen synthase kinase-3β
HDL-C	High-Density Lipoprotein Cholesterol
HLP	Hyperlipidemia
HTN	Hypertension
LPSs	Lipopolysaccharides
LV	Left Ventricle
METs	Metabolic Equivalents
MIND	Mediterranean–DASH Intervention for Neurodegenerative Delay
MUFAs	Monounsaturated Fatty Acids
NF-κB	Nuclear Factor kappa B
NFTs	Neurofibrillary Tangles
NO	Nitric Oxide
PUFAs	Polyunsaturated Fatty Acids
RCTs	Randomized Controlled Trials
RAGE	Receptors for Advanced Glycation End
ROS	Reactive Oxygen Species
SCFAs	Short-Chain Fatty Acids
SORL1	Sortilin-Related Receptor
T2DM	Type 2 Diabetes Mellitus
TCIM	Transcriptional and Immune-response Modulator
TG	Triglyceride
VLDL	Very Low-Density Lipoprotein
Wnt/β-catenin	wingless-related integration site/beta-catenin
